# Constructing a Genome-Wide LD Map of Wild *A. gambiae* Using Next-Generation Sequencing

**DOI:** 10.1155/2015/238139

**Published:** 2015-09-03

**Authors:** Xiaohong Wang, Yaw A. Afrane, Guiyun Yan, Jun Li

**Affiliations:** ^1^Department of Chemistry and Biochemistry, University of Oklahoma, Norman, OK 73019, USA; ^2^Centre for Global Health Research, Kenya Medical Research Institute, Kisumu 40100, Kenya; ^3^Program in Public Health, University of California, Irvine, CA 92697, USA

## Abstract

*Anopheles gambiae* is the major malaria vector in Africa. Examining the molecular basis of* A. gambiae* traits requires knowledge of both genetic variation and genome-wide linkage disequilibrium (LD) map of wild* A. gambiae* populations from malaria-endemic areas. We sequenced the genomes of nine wild* A. gambiae* mosquitoes individually using next-generation sequencing technologies and detected 2,219,815 common single nucleotide polymorphisms (SNPs), 88% of which are novel. SNPs are not evenly distributed across* A. gambiae* chromosomes. The low SNP-frequency regions overlay heterochromatin and chromosome inversion domains, consistent with the lower recombinant rates at these regions. Nearly one million SNPs that were genotyped correctly in all individual mosquitoes with 99.6% confidence were extracted from these high-throughput sequencing data. Based on these SNP genotypes, we constructed a genome-wide LD map for wild* A. gambiae* from malaria-endemic areas in Kenya and made it available through a public Website. The average size of LD blocks is less than 40 bp, and several large LD blocks were also discovered clustered around the* para* gene, which is consistent with the effect of insecticide selective sweeps. The SNPs and the LD map will be valuable resources for scientific communities to dissect the* A. gambiae* genome.

## 1. Background

Malaria, a mosquito-transmitted disease caused by parasites of the genus* Plasmodium*, leads to as many as 300 million clinical cases per year [1]. Of these, approximately one million die from malaria, with 75% of the deaths occurring in African children. Human malaria parasites are transmitted by anopheline mosquitoes, of which* Anopheles gambiae *is the most prevalent vector in Africa.

Genetic variation in mosquito populations affects the mosquitoes' susceptibility to* P. falciparum* infection [2–4], insecticide resistance [5–8], and other traits of interest. Determining the molecular basis for these and other important mosquito traits requires knowledge of genome-wide genetic variation and high-resolution linkage maps in wild* A. gambiae* populations from malaria-endemic areas. The currently available* A. gambiae* SNPs in the NCBI database dbSNP mainly derive from laboratory mosquito colonies [9, 10]. Sampling a small set of genes [11] or SNPs [12] in field-collected samples indicated low linkage disequilibrium (LD) in* A. gambiae* populations. However, this result means neither that neighboring SNPs are not linked, nor that large LD blocks in the* A. gambiae* genome do not exist. Therefore, it is still critical to define the extent of genome-wide genetic variation and linkage information in* A. gambiae* populations from malaria-endemic areas. Recent advances in sequencing technologies and bioinformatics make it economically feasible for a single research laboratory to detect genome-wide SNPs and to construct an* A. gambiae* LD map using wild-derived mosquitoes. Depending on needs, the current available software such as Haploview [13] allows the users to easily generate an interactive haplotype map for a whole genome or a certain genomic region based on a set of genotypes or LD map.

In this study, we collected wild* A. gambiae* from Kenya, sequenced individual mosquitoes with Illumina sequencing technologies, developed novel pipelines to detect SNPs, constructed an* A. gambiae* LD map, and established a computer server to present the data to the public. Notably, the consistence between our data and experimental findings supports the accuracy of this resource and demonstrates the advantages of the SNPs and LD map.

## 2. Results

### 2.1. Detecting SNPs in Wild* A. gambiae* Mosquitoes

**Figure 1:**
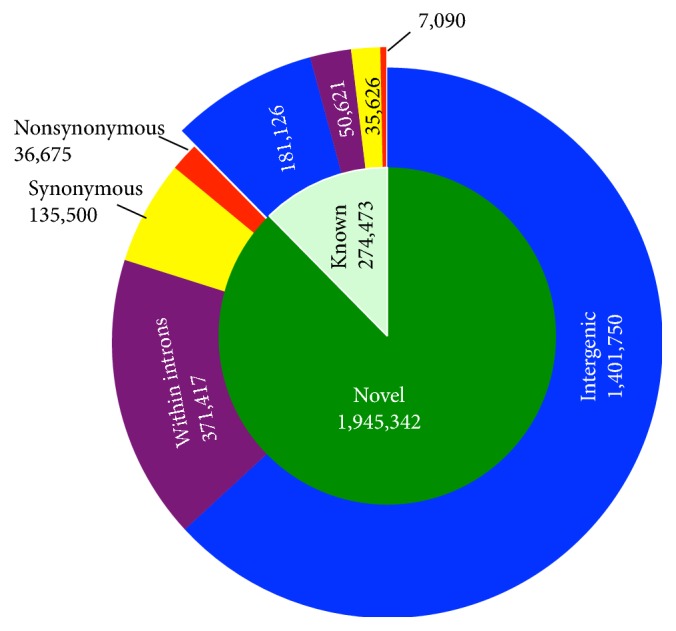
Types of common SNPs. About 87.6% of newly detected common SNPs from wild* A. gambiae* are novel. The types of common SNPs were determined based on their positions on the genome (intergenic: blue; within introns: purple; synonymous: yellow; nonsynonymous: red). SNPs within exons were further classified into synonymous and nonsynonymous. About 2% of SNPs changed protein sequences. The number of SNPs is shown in each category.

To detect common SNPs (frequencies > 5%) in wild* A. gambiae* populations, individual genomic DNA from nine randomly selected wild* A. gambiae *mosquitoes recovered from highland areas around Kisumu was studied. DNA from each mosquito was sequenced individually after the samples were confirmed to be* A. gambiae*. Each sequence read was 100 bp long. The average sequencing coverage of the whole genome for each individual was greater than 36.1-fold. These reads were mapped onto the* A. gambiae* reference genome [9]. More than 1.6 million SNPs were detected in each individual mosquito according to the aligned short reads. Among the detected SNPs, 2,219,815 common SNPs were detected in more than one mosquito, and about 4,911,116 unique SNPs were detected in only one mosquito. The SNP-frequency is about one SNP per 33 bp, which is consistent with previous reports [11, 14]. To check the detection accuracy, a randomly selected set of SNPs was verified using a graphical user interface (http://omics.ou.edu/AgHapMap). The results indicated a low error rate of less than 0.1%. Notably, as many as 87.6% of the newly discovered SNPs are novel, compared to SNPs in dbSNP (NCBI, release 125) [15] that were mainly from mosquito colonies maintained in laboratories. This suggests that these SNPs are useful resources to study* A. gambiae* in the field. Among the novel common SNPs, 36,675 (1.9%) are nonsynonymous, 135,500 (7.0%) are synonymous, 371,417 (19.1%) are within intron regions, and the others (1,401,750, 71.3%) are at intergenic regions (Figure 1). The SNP type distribution for novel SNPs is similar to the known SNPs.

**Figure 2:**
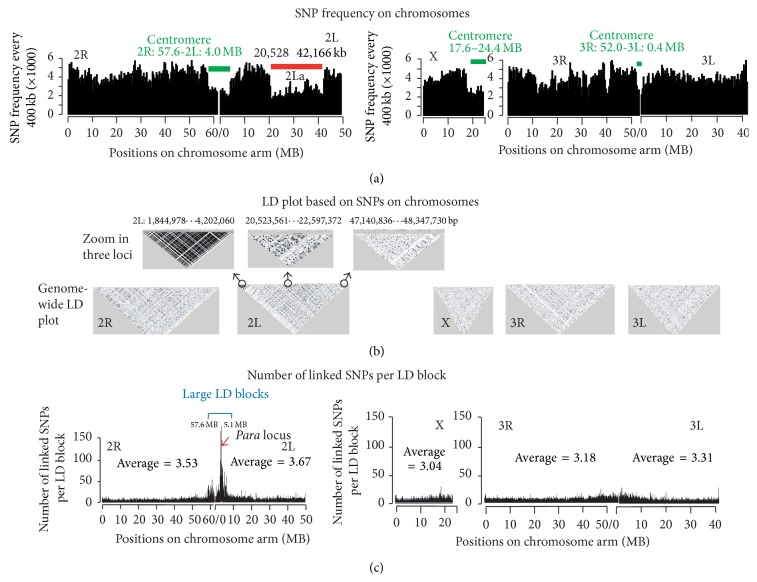
Genome-wide SNP-frequency, LD plots, and number of SNPs per LD block. (a) shows the SNP-frequency (per 400 kb) on* A. gambiae* chromosomes. The genomic regions (>1 MB) with less than 1 SNP per 150 bp were labeled with green and red lines. (b) shows LD plot of five chromosome arms and zoom in of three particular regions (high LD, chromosome inversion region, and other regions) to illustrate the genome-wide linkage map in detail. (c) The number of SNPs per LD block on chromosomes. The *x*-axes of (a) and (c) correspond to the same positions.

We identified that four large genomic regions, for example, 2R:57.6 MB-2L:4.0 MB, 2L:20.5–42.2 MB, X:17.6–24.4 MB, and 3R:52.0 MB-3L:0.4 MB, have much lower frequencies of SNPs, compared to other regions (Figure 2(a)). As expected, three of these regions (labeled with green lines in Figure 2(a)) are around centromeres on chromosome 2, X and 3, consistent with characteristics of heterochromatic regions [16]. Strikingly, a region on chromosome arm 2L from 20.5 MB to 42.2 MB (labeled with red line in Figure 2(a)) also exhibited low frequencies of SNPs and overlaid a chromosomal inversion called 2La [17]. We karyotyped the inversion of 2La in the nine mosquitoes using PCR [18], and the results show that four mosquitoes were 2L+^a^/2L+^a^ and five were 2La/2La. The 2L chromosomal inversion region always had fewer SNPs than other genomic regions, regardless of mosquito karotypes. The consistence of the low recombinant rate at heterochromatic regions and chromosomal inversion to the SNP distribition partially validates the SNPs genome-widely.

### 2.2. The LD Map in Wild* A. gambiae* Populations from Malaria-Endemic Areas in Kenya

**Figure 3:**
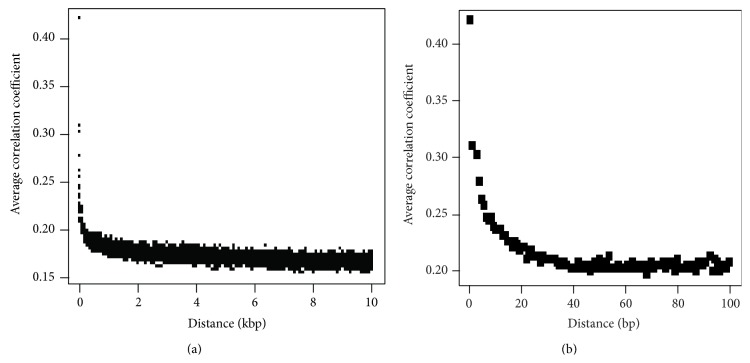
LD decays rapidly as the distance of SNPs increases. (a) displays the relationship between correlation coefficient and SNP distance from 0 to 10 kb. (b) shows the relationship between correlation coefficient and SNP distance from 0 to 100 bp, which clearly shows that average genome-wide LD size is less than 40 bp.

We next extracted the SNP genotypes of individual mosquitoes based on the high-throughput short read sequences as described in the Methods section. Out of 2,219,815 common SNPs, 785,687 SNPs (one SNP per every 293 bp genome-wide) were reliably genotyped at 99.6% confidence in nine mosquitoes. The correlation coefficient among SNPs was calculated by using Haploview software [13]. As shown in Figure 3(a), the average coefficient of determination over distance between SNPs decreases rapidly. Moreover, when the distance between two SNPs is greater than 40 bp, the linkage relation between SNPs is nearly random (Figure 3(b)).

Although the average LD size in* A. gambiae* is very short (Figure 3), which is consistent with previous reports [11, 12], our genome-wide, high-throughput LD analysis also identified regions with very large LD blocks (Figures 2(b) and 2(c)). For instance, the locus at 2L: 1.8 MB–4.2 MB contains four large LD blocks, and the average LD size at locus 2L: 20.5 MB–22.6 MB is apparently greater than the LD size at locus 2L: 47.1 MB–48.3 MB (Figure 2(b)). To accurately quantitate the linkage relationship between neighboring SNPs efficiently, we calculated the number of SNPs per LD block. According to the plot of coefficient of determination versus distance (Figure 3(b)), two neighboring SNPs were considered to be linked if their correlation coefficient was greater than 0.25. Although most of LD blocks contained less than 3 SNPs, several very large LD blocks with more than 50 SNPs were clustered at a locus on chromosome 2 (2R:57.6 MB-2L:5.1 MB), indicated by a blue line in Figure 2(c). Detailed analysis of the genes within this genomic region clustering large LD blocks found that the* para* gene (AGAP004707) was at the center (2L, 2.4 MB) of the large LD clusters (Figure 2(c)). All nine sequenced mosquitoes from malaria-endemic areas at highland areas around the Kisumu district in western Kenya are homozygous for the insecticide resistant allele cytosine, which forms the code of “TCA” and encodes amino acid serine in the voltage-gated sodium channel [19]. The biological reason of this locus validates the new LD map and demonstrates the usability of the LD map.

We further verified the* A. gambiae* LD map experimentally. Two pairs of SNPs within neighboring genes were genotyped in 22 randomly selected female wild-derived* A. gambiae*: SNP at chromosomal arm 2L, 39,852,810 bp within gene AGAP006906 versus SNP at chromosomal arm 2L, 39,966,795 bp within gene AGAP006914, and SNP at chromosomal arm 2L at 41,165,983 bp within gene AGAP007031 versus SNP at chromosomal arm 2L at 41,246,582 bp within gene AGAP007032. The results (Table 1) showed that the coefficient of determination between pairs of SNPs was less than 0.05, experimentally validating the computational LD results from high-throughput sequencing.

### 2.3. Public Web to Integrate Aligned Reads, SNPs, LD Map, and Genome Annotation

**Figure 4:**
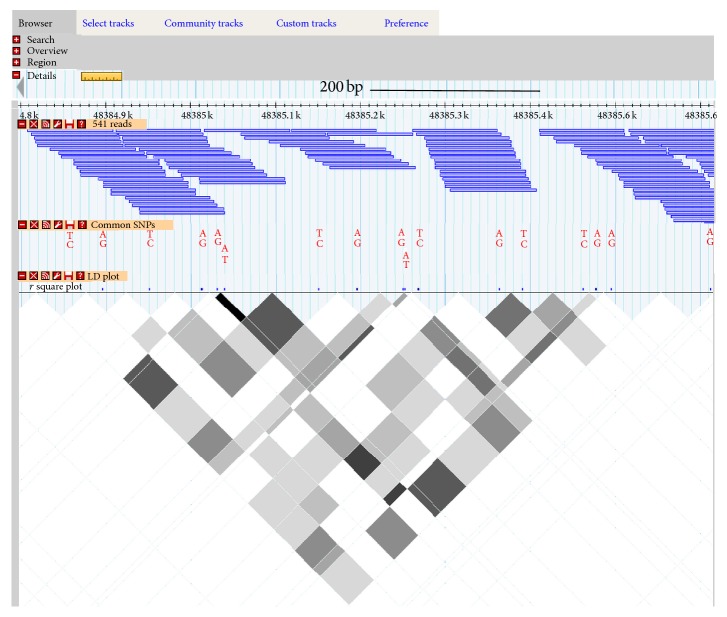
Screenshot of the Web interface to display SNPs, reads, LD Map, and external genome annotation. Users can obtain reads, SNPs, and LD at http://omics.ou.edu/AgHapMap. The sequences and alignments of reads can be viewed in detail by highlighting and zooming in. Data tracks from internal and external databases, which are not shown in this screenshot, can be integrated by selecting through the Tab of “Select Tracks.”

To make these valuable data available to the scientific community, we established a computer server and constructed databases and a Web interface to visualize the SNPs, LD map, short reads, and detailed alignments, along with internal and external genome annotations. The Website is accessible through http://omics.ou.edu/AgHapMap. After access, users can click on the tab “Select Tracks” to select the data that they are interested in and click on “Browser” to see actual data. To zoom in on a particular region, they can highlight the region and click on “zoom in.” Figure 4 shows the screen shot of this server.

## 3. Discussion

Genetic variation and LD maps are two important resources that enable identification of genetic mutants associated with traits of interest in populations. However, it is impractical to detect genetic variation and build a genome-wide LD map for all species with traditional approaches, for example, by surveying a set of genetic markers in populations. To overcome these limitations, we extracted and sequenced genomic DNA from individual mosquitoes with high-throughput sequencing technologies. Next, we developed a pipeline to obtain more than two million SNPs. Importantly, the majority (88%) of our SNPs from wild-derived* A. gambiae* are novel, which will help the community to address the molecular mechanisms of trait determination, as well as potentially reconciling discrepancies when comparing results obtained from laboratory mosquitoes versus field isolates [20]. Furthermore, we developed a novel computational approach to genotype nearly one million SNPs in individual mosquitoes solely based on our high-throughput sequencing data. Traditional approaches for genotyping individuals requires a* priori* knowledge of genetic markers, and it is tedious to genotype each genetic marker in individuals using hybridization-based methods or PCR-based approaches. In this report, we combined SNP discovery with SNP genotyping using a new computational pipeline, making the process both more efficient and cost-effective. Using our high-throughput sequencing data and our computational pipeline, we have assembled the first genome-wide LD map of* A. gambiae, *using wild-derived mosquitoes from malaria-endemic areas in western Kenya. We also report here that the newly detected genetic variation and LD map have been made freely and easily accessible to the public through the Internet. Notably, our approach and pipeline are applicable to generate LD maps of other biologically, agriculturally, and medically important mosquito species.

As mentioned above, it is well-known that the genetic variation and LD maps are important for association studies to analyze wild mosquitoes [2, 3], and we have also demonstrated their utility in our previous publication [4]. The interaction among multiple genetic variation within multiple genes also contributes to a complex trait [21]. Here, we highlight additional and powerful applications of genetic variation and the LD map to investigate aspects of mosquito biology in nature.

SNP distribution identified four large genomic regions harboring unusually low frequencies of SNPs, three of which localized to centromeres. Although this observation was expected, given that the genomic regions around centromeres have lower recombination rates than other loci and DNA recombinant rates and SNP density are positively correlated [22], we also identified a fourth large locus with lower SNP-frequency on 2L at 20.5–42.2 MB. Notably, the fourth locus was also associated with lower recombination rates because this region contains a chromosomal inversion [17], and chromosomal inversions are known to inhibit DNA recombination [23]. We karyotyped the inversion forms of 2La in individual* A. gambiae* to investigate whether our sample contained a single karyotype form that causes the lower SNP-frequency at this region. Our results show that the lower SNP-frequency did not associate with any particular karyotype form of chromosomal inversion of 2La. Apparently, the consistence between SNP distribution and genetic data validates the detected SNPs genome-widely and demonstrates their usability.

Regarding our* A. gambiae* LD map, a previous survey of a limited set of genes (*n* = 4) [11] or SNPs (*n* = 1,536) [12] suggested low LD (<200 bp) in* A. gambiae* populations. Our data are consistent with those reports. However, here we extend these observations and show that the average LD size in* A. gambiae* populations in western Kenya is less than 40 bp. It is worth noting that our new results additionally provide a genome-wide LD map of nearly one million genetic markers. Furthermore, the LD map reveals a genomic locus on chromosome 2 (2R: 57.6 MB-2L: 5.1 MB) that is clustered with larger LD blocks. Analyses of the genes within this region identified that the* para* gene is at the center of this locus. The* para* gene encodes a voltage-gated sodium channel (VGSC) that is the target molecule of common insecticides such as pyrethroids [24]. The well-known* kdr* mutations, which change codon 1014 from leucine to serine or phenylalanine within the* para* gene coding region, confer insecticide resistance [25–27]. Indeed, all mosquitoes that we sequenced (*n* = 9) harbored the resistance allele (1014S) instead of the wild type allele (1014L). It is well known that the use of insecticides remains the traditional approach to combating the spread of malaria [28]. The molecular target of common insecticides such as pyrethroids and dichlorodiphenyltrichloroethane (DTT) is VGSC [24, 29]. DDT and pyrethroids were used globally, including Kenya [30], and caused an insecticide-driven selective sweeping in western Kenya. These data are consistent with insecticide resistance bioassays in the field [19, 31] where our mosquitoes were sampled. Rapid rise of* kdr* mutation frequency and even fixation over the past decade when pyrethroid insecticides have been used extensively in Africa suggest the importance of this mechanism in the process of pyrethroid resistance. On the other hand, given the fixation of* kdr* mutations in many* A. gambiae* populations, metabolic detoxification is becoming an increasingly important resistance mechanism. Clearly, vector insecticide resistance is an outstanding issue in the control and prevention of vector-borne diseases as supported by our data and other reports [32, 33]. Collectively, the larger LD around the* para* gene validates our LD map and demonstrates an application of using our LD map to detect genomic regions under selection pressure.

In conclusion, we collected and sequenced wild* A. gambiae* mosquitoes from malaria-endemic areas in Kenya using next-generation sequencing technology and developed a pipeline to analyze SNPs and genotypes. More than 2 million common SNPs were identified in wild* A. gambiae* populations, and 785,687 SNPs were genotyped in nine mosquitoes. Using these data, we constructed the first genome-wide* A. gambiae* LD map, which will serve as a powerful and useful resource to dissect the mosquito genome. The consistence between our data and previous findings supports the accuracy of this resource.

## 4. Methods

### 4.1. Sampling Wild* A. gambiae*

Collecting and rearing mosquitoes were performed as described previously [4]. In brief,* A. gambiae* larvae were collected from natural habitats (>10 meters distance between any two habitats) in highland areas around the Kisumu district of Kenya where malaria is hyperendemic. More than half of mosquito larvae were successfully reared to adults in an insectary at the Kenya Medical Research Institute. The resulting 3–5-day post-emergence female mosquitoes were used for experiments. It is worth noting that only the female wild-derived mosquitoes that fed on human blood through membrane feeding were further analyzed in this study. Genomic DNA was extracted from 7-day post-blood-fed mosquitoes using DNAzol (Life Technologies, Grand Island, NY, USA). The individual mosquito species was confirmed by the rDNA-PCR method [34].

### 4.2. Sequencing Individual* A. gambiae* Genomes and Detecting SNPs

Genomic DNA from individual mosquitoes was sheared to construct a DNA library with fragment lengths of about 300 bp, and both sides of each DNA fragment were sequenced in lengths of 100 bp. These reads were mapped to the* A. gambiae* reference genome (assembly version AgamP3) using the short oligonucleotide analysis package (SOAP) [35]. SOAP used the seed-and-hash algorithm to align high-throughput sequences onto the reference genome accurately and efficiently. To focus on the SNP detection, we turned off the option for gaps, for example, “soap –a leftReads –b rightRead –D referenceGenome.index –o AlignedFile.txt –m 50 –x 550 –g 0.” The alignments for each chromosome were then extracted using the linux command “grep,” for example, “grep X AlignedFile.txt > X.align,” followed by sorting the output based on alignment position on chromosome, for example, “sort –k9 –n X.align > X.align.sort.” Finally, the “soapsnp” program in the SOAP package was used to detect nucleotide variation at each position, for example, “soapsnp –i X.align.sort –d reference_genome_seq.fasta –o SNPonX.” At each genome position, we extracted the SNPs that had at least one uniquely mapped read for best base and at least one uniquely mapped read for second best base (phrep score > 30) [36]. The nucleotides that are different from the reference sequence were also extracted into the SNP set. To obtain common SNPs, we removed the SNPs that (1) were detected in only one mosquito and (2) were identical in all nine mosquitoes (they were detected because they were different from the reference genome). Finally, we checked the error rate by using our web interface. We randomly selected SNPs three times with 100 SNPs each time. Then we manually examined the aligned sequence reads to the reference genome sequences to count the true positives.

### 4.3. Genotyping the SNPs in Nine Individual Mosquitoes Based on High-Throughput Sequencing Data

For each detected SNP, we checked all reads in each individual mosquito regardless of its sequencing quality score (Phred score) [36]. If two alleles for an SNP were detected in reads from one* A. gambiae* individual, a heterozygous genotype was assigned to that individual for that genome position. However, for each homozygous SNP genotype, the number of reads hitting that position was counted using the information obtained through the program “soapsnp” from the SOAP package [35]. If a homogenous SNP genotype was supported by at least eight reads from that mosquito, it was kept for LD map analysis, because the *P* value of missing a heterozygous allele is less than 0.004 based on the binomial distribution. The genotypes of these SNPs were applied to calculate the LD among SNPs by using the software Haploview [13]. Two neighboring SNPs with a correlation coefficient greater than a threshold (e.g., 0.25 in Figure 3(b)) were treated as linked SNPs in one LD block.

### 4.4. Genotyping a Particular Set of SNPs in Individual Mosquitoes Using PCR Followed by the Sanger Sequencing

We cloned two pairs of neighboring genes: AGAP006906 versus AGAP006914 and AGAP007031 versus AGAP007032 to verify the LD map. The sequences around nonsynonymous SNPs were cloned from 22 randomly selected female wild-derived* A. gambiae* by PCR with primers shown in Table 2. The PCR products were purified using QIAGEN PCR purification kits. The purified DNA fragments were sequenced with one PCR primer using Sanger approach. The sequencing trace files were displayed using software 4Peaks (http://nucleobytes.com/), and the SNPs were read manually.

### 4.5. Visualizing SNPs, Short Reads, Genome Annotation, and the HapMap through an Integrated Web Interface

**Figure 5:**
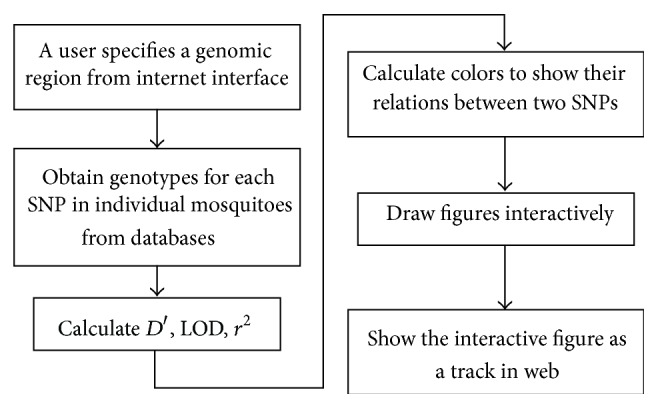
Protocol to construct a Web server to display* A. gambiae* LD Map.

The individual short reads, the sequence alignment to the reference genome, and the SNPs were compiled into databases as instructed by Gbrowse and displayed as tracks [37]. In brief, “samtools” (Sequence Alignment/Map tools) software downloaded from http://samtools.sourceforge.net/ was used to transform a data file from one format to another. The “samtool” was also used to import data into Gbrowse required databases. To generate these databases, the reference genome sequence was indexed (e.g., “samtools faidx ReferenceSequence.fa”), and then the data were imported into databases (e.g., “samtool import ReferenceSequence.fa.fai”). The alignment files that contain short reads aligned to the reference sequence were sorted and indexed (e.g., “samtool sort aligmentFile.bam aligmentFile.sorted.bam” and “samtool index aligmentFile.sorted.bam”) sequentially. Finally, the sorted and indexed alignment files were imported into databases (e.g., “samtool import aligmentFile.sorted.bam.bai”). SNP data were stored in a mysql database with a single table that was created with this command: “CREATE TABLE Agam_common_snps_position (snp varchar(10) NOT NULL, alleles varchar(4) NOT NULL, chr ENUM (‘2L',‘2R',‘3L',‘3R',‘X'), pos int(10) unsigned NOT NULL default ‘0', Ag541 char(2), Ag544 char(2), Ag545 char(2), Ag551 char(2), Ag553 char(2), Ag564 char(2), Ag565 char(2), Ag566 char(2), Ag567 char(2), PRIMARY KEY (chr,pos), KEY chr (chr), KEY pos (pos));”. Each data set was displayed as a track (also known as a “plug-in”) through Gbrowse. For instance, the genome annotation, including gene structure predictions in our internal databases (ReAno) [10] and external databases of https://www.vectorbase.org/ [38], was integrated as two tracks on the Web. The correlation-coefficient values among SNPs were constructed and integrated into the Web interface. To display an interactive graphic of the linkage map through the Internet, we constructed the server using a protocol as shown in Figure 5. In brief, the server obtains the interactive coordinators of SNPs and SNP genotypes from the databases and calculates the LD (*D*′), logarithm of odds (LOD), and coefficient of determination (*r* ^2^) for each pair of SNPs interactively. Based on a user's HapMap configuration (or default), the server calculates the colors and displays it as a plug-in track on the Web interface. Using downloaded Haploview software [13], users can easily generate an interactive haplotype map at areas of interest (such as large LD blocks) by highlighting the LD blocks with the mouse under the “LD plot” tab and then clicking on the tab “Haplotypes.” The phased haplotypes of the highlighted blocks will then be displayed. The constructed HapMap may need further experimental validation.

## Figures and Tables

**Table 1:** Correlation coefficient between nonsynonymous SNPs of two pairs of neighboring genes.

	Based on HT (9 individuals)	Based on clone (22 individuals)
AGAP006906 versus AGAP006914	0.156	0.009
AGAP006914 versus AGAP007031	0.156	0.02
AGAP007031 versus AGAP007032	0.044	<0.001

AGAP006906, SNP position (bp), 39852810; AGAP006914, SNP position, 39966795; AGAP007031, SNP position, 41165983; AGAP007032, SNP position, 41246582. HT: high-throughput sequencing data. Clone: PCR fragments from individual mosquitoes.

**Table 2:** Primers to clone two pairs of neighboring genes.

AGAP006906	Forward	5′-CGGAGGCACACACCATCA-3′
	Reverse	5′-GCGAAACTCCAGATACAGCA-3′

AGAP006914	Forward	5′-CAACTGCTGGCCAAAGGAC-3′
	Reverse	5′-GTCCTTTGGCCAGCAGTTG-3′

AGAP007031	Forward	5′-GGCTCGAAGTCCGATTACA-3′
	Reverse	5′-GTCGGCACAGTCGTGGTA-3′

AGAP007032	Forward	5′-ATAACCATGCGGAGAGTGTG-3′
	Reverse	5′-CCGTTCGATTTCCTCCTG-3′
